# Supplementary material for: Asynchronous mixing of kidney progenitor cells potentiates nephrogenesis in organoids
Source: Commun Biol. 2020 May 11;3:231. doi: 10.1038/s42003-020-0948-7 (PMC7214420; doi:10.1038/s42003-020-0948-7)
Supplement: Supplementary file 1 — Supplementary Information [file 42003_2020_948_MOESM1_ESM.pdf]

SUPPLEMENTARY INFORMATION

Supplementary Figures

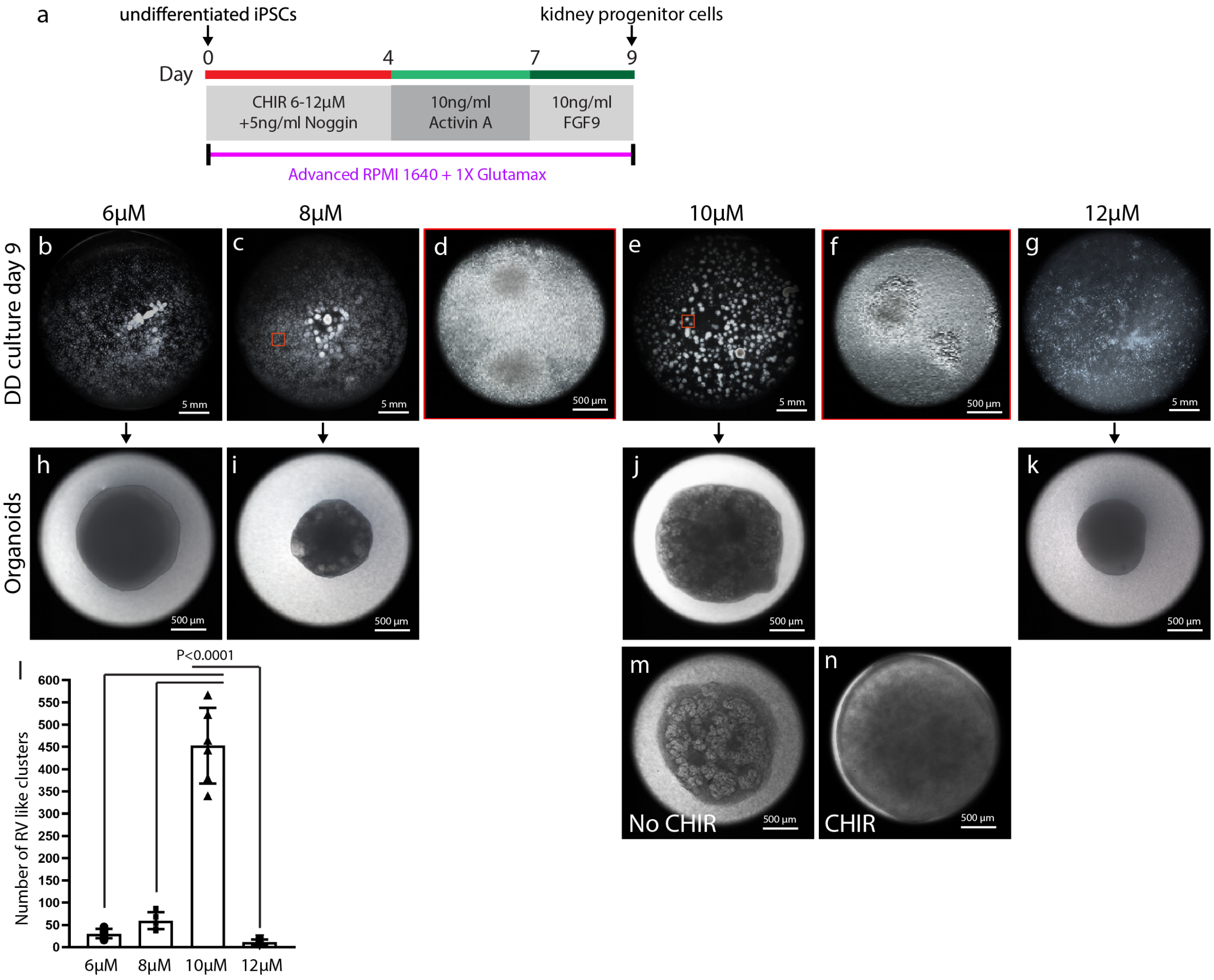

Supplementary Figure 1: CHIR dose adjustment for directed differentiation of WTC11 human iPSCs. **(a)** The directed differentiation procedure showing the period between initiation of directed differentiation (day 0) and 4 days during which time CHIR was titrated. Stereo microscope images of cell culture plates on day 9 showing clusters of epithelializing cells following treatment with **(b)** 6 μM, **(c)** 8 μM. **(d)** Magnified view of sparse cell clusters. **(e)** 10μM. **(f)** Magnified view of dense cell clusters with epithelial morphology, and **(g)** 12 μM CHIR. **(h-k)** Stereo microscope images showing kidney organoids generated by aggregation and culture at the air-liquid interface as shown in Figure 1 for each directed differentiation. **(l)** Clusters with epithelial morphology were counted. Clusters in three random fields were counted in each well of six-well plate. Values calculated from n=3 independent biological replicates and expressed as mean±s.d. One-way ANOVA with Tukey's multiple-comparisons test was applied to calculate P value. (**m**,**n**) Direct comparison of organoids from directed differentiations using 10μM CHIR either allowed to spontaneously differentiate (**m**) or treated with 3μM CHIR for 48 hours following aggregation (**n**).

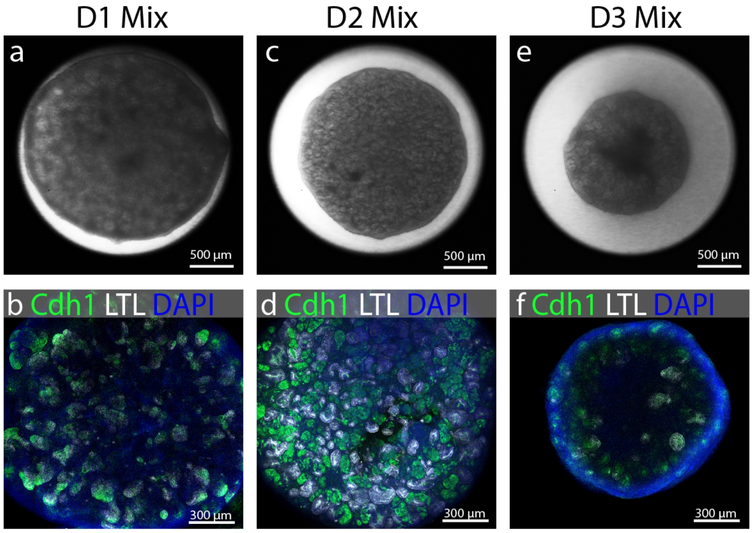

Supplementary Figure 2: Determination of time point for heterochronic recombination. Kidney progenitors derived from directed differentiation on day 9 were aggregated on air liquid interface and were recombined with a fresh batch of kidney progenitors after 1 day (D1), 2 days (D2) and 3 days (D3). **(a, c and e)** Stereo microscope images showing organoid size and complexity. **(b, d and f)** Immunostaining with Cdh1+ to mark distal epithelia, and LTL to mark proximal. DAPI stains nuclei.

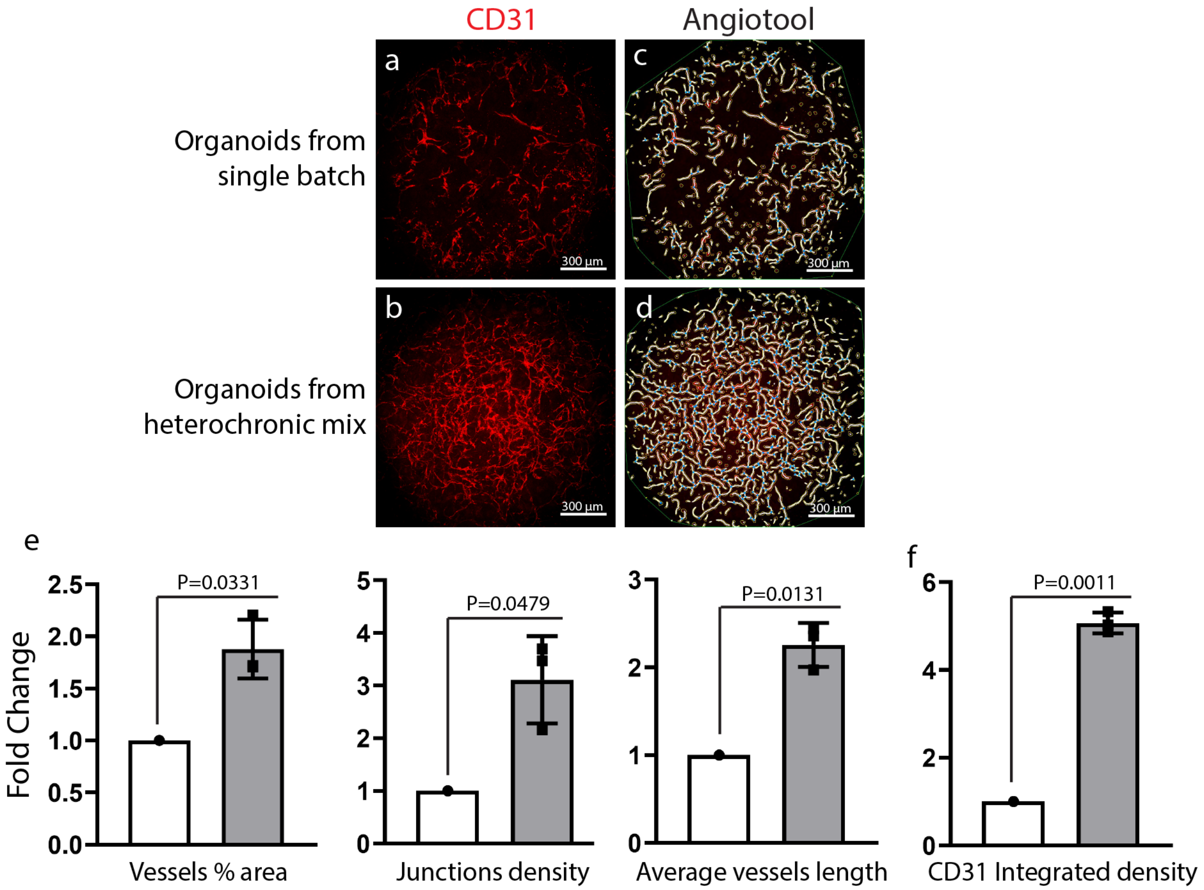

Supplementary Figure 3: Comparison of endothelial networks in organoids derived from single batches of directed differentiation cells or heterochronic mixes. Immunofluorescence image showing CD31+ endothelial network in organoid generated from **(a)** single batch and **(b)** heterochronic recombination. **(c-d)** Maximal projections of confocal Z-stacks quantified using Angio tool software. White lines depict endothelial networks, blue dots show junctions, yellow lines showing edge of vascular networks and thin green lines showing total area over which organoid was quantified. **(e)** Fold change in vessel % area, junction density and average vessel length was calculated from single mix (open bars) and heterochronic mix organoids (gray bars). Endothelial networks in three random fields were counted in each organoid. Values calculated from n=3 independent biological replicates and expressed as mean±s.d. Unpaired t test with Welch's correction was applied to calculate P value. **(f)** Fold change in CD31 integrated density was quantified by Image J in single mix (open bars) and heterochronic mix organoids (gray bars). CD31 integrated density in three random fields were counted in each organoid. Values calculated from n=3 independent biological replicates and expressed as mean±s.d. Unpaired t test with Welch's correction was applied to calculate P value.

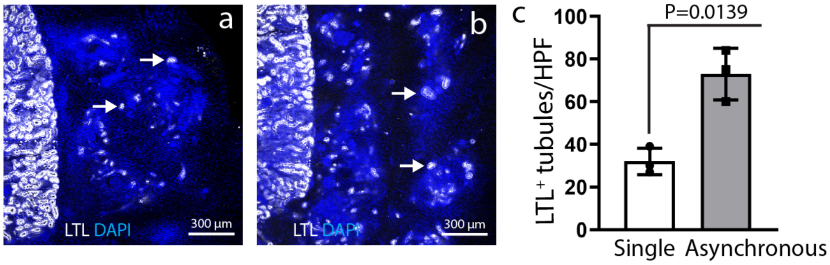

Supplementary Figure 4: Comparison of engraftment of single batch versus heterochronic organoids. Organoids were prepared either from single batches (**a**) or heterochronic mixes (**b**) of directed differentiation cells. LTL was used to detect proximal tubule structures (arrows). (**c**) Quantification of LTL structures in grafts. Values calculated from n=3 independent biological replicates and expressed as mean±s.d. Unpaired t test with Welch's correction was applied to calculate P value.

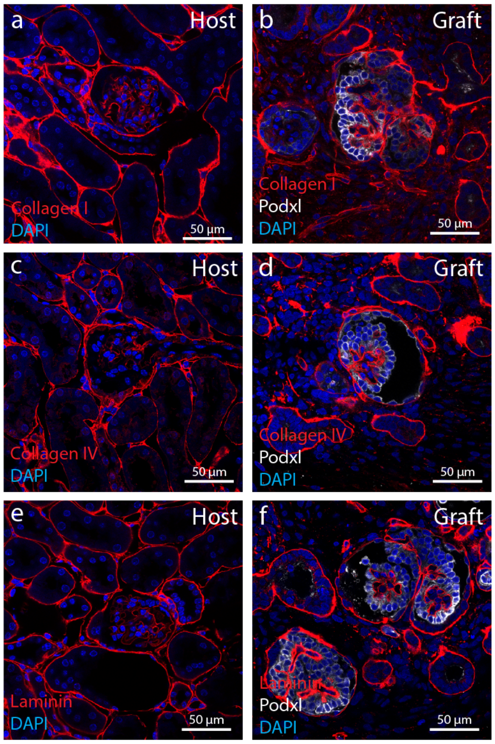

Supplementary Figure 5: Glomerular basement membrane in the engrafted organoids. (a-c) Engraftment of organoids resulted in more mature nephron structures and presence of Collagen I^+, Collagen IV^+ and $Laminin^+$ glomerular basement membrane surrounded by $Podxl^+$ podocytes in presumptive glomerulus.

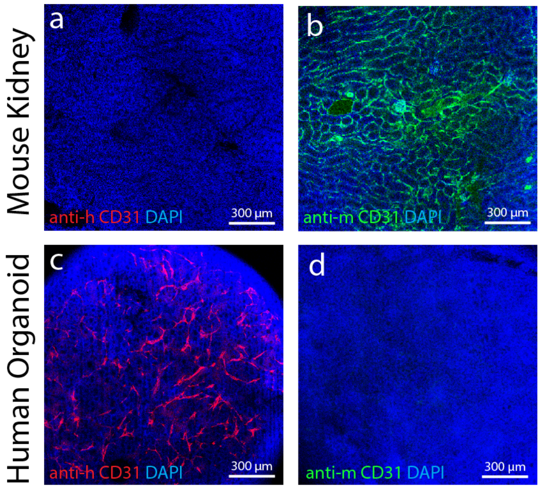

Supplementary Figure 6: Species-specificity of CD31 antibodies. Adult mouse kidney stained with anti-human CD31 shows no signal (**a**), whereas staining with anti-mouse CD31 shows blood vessel staining (**b**). Human organoid stained with anti-human CD31 shows strong signal (**c**), whereas staining with anti-mouse CD31 shows no signal (**d**).

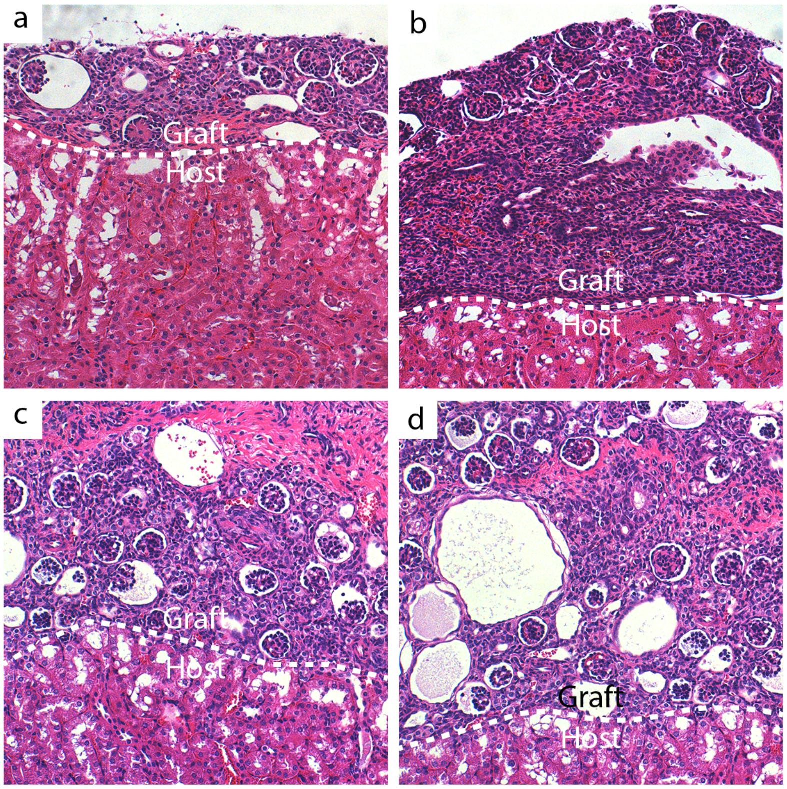

Supplementary Figure 7: Engraftment of E15.5 mouse kidney under the capsule of adult NSG mouse kidneys. Examples of histology show extensive stromal accumulation in the graft tissue (**a-d**).
