# Supplementary material for: Asynchronous mixing of kidney progenitor cells potentiates nephrogenesis in organoids
Source: Commun Biol. 2020 May 11;3:231. doi: 10.1038/s42003-020-0948-7 (PMC7214420; doi:10.1038/s42003-020-0948-7)
Supplement: Supplementary file 3 — Description of Additional Supplementary Files [file 42003_2020_948_MOESM3_ESM.pdf]

Description of Additional Supplementary Files

File Name: **Supplementary Data 1**
Description: Source data file
