# Supplementary material for: Asynchronous mixing of kidney progenitor cells potentiates nephrogenesis in organoids
Source: Commun Biol. 2020 May 11;3:231. doi: 10.1038/s42003-020-0948-7 (PMC7214420; doi:10.1038/s42003-020-0948-7)
Supplement: Supplementary file 5 — Reporting Summary [file 42003_2020_948_MOESM5_ESM.pdf]

nature research

Corresponding author(s): Leif Oxburgh
Last updated by author(s): Feb 20, 2020

Reporting Summary

Nature Research wishes to improve the reproducibility of the work that we publish. This form provides structure for consistency and transparency in reporting. For further information on Nature Research policies, see Authors & Referees and the Editorial Policy Checklist.

Statistics

For all statistical analyses, confirm that the following items are present in the figure legend, table legend, main text, or Methods section.

n/a	Confirmed	
☐	☒	The exact sample size (*n*) for each experimental group/condition, given as a discrete number and unit of measurement
☐	☒	A statement on whether measurements were taken from distinct samples or whether the same sample was measured repeatedly
☐	☒	The statistical test(s) used AND whether they are one- or two-sided<br>*Only common tests should be described solely by name; describe more complex techniques in the Methods section.*
☒	☐	A description of all covariates tested
☐	☒	A description of any assumptions or corrections, such as tests of normality and adjustment for multiple comparisons
☐	☒	A full description of the statistical parameters including central tendency (e.g. means) or other basic estimates (e.g. regression coefficient) AND variation (e.g. standard deviation) or associated estimates of uncertainty (e.g. confidence intervals)
☐	☒	For null hypothesis testing, the test statistic (e.g. *F*, *t*, *r*) with confidence intervals, effect sizes, degrees of freedom and *P* value noted<br>*Give P values as exact values whenever suitable.*
☒	☐	For Bayesian analysis, information on the choice of priors and Markov chain Monte Carlo settings
☒	☐	For hierarchical and complex designs, identification of the appropriate level for tests and full reporting of outcomes
☒	☐	Estimates of effect sizes (e.g. Cohen's *d*, Pearson's *r*), indicating how they were calculated

Our web collection on statistics for biologists contains articles on many of the points above.

Software and code

Policy information about availability of computer code

Data collection	Standard bright-field, stereo and confocal microscopes, vivaCT 40 scanner were used to collect data.
Data analysis	Statistical analyses were performed using GraphPad Prism 5, ImageJ or Angio tool software.

For manuscripts utilizing custom algorithms or software that are central to the research but not yet described in published literature, software must be made available to editors/reviewers. We strongly encourage code deposition in a community repository (e.g. GitHub). See the Nature Research guidelines for submitting code & software for further information.

Data

Policy information about availability of data

All manuscripts must include a data availability statement. This statement should provide the following information, where applicable:

- Accession codes, unique identifiers, or web links for publicly available datasets
- A list of figures that have associated raw data
- A description of any restrictions on data availability

No publicly available datasets were used. The data generated in this study is available from the corresponding author on reasonable request.

Field-specific reporting

Please select the one below that is the best fit for your research. If you are not sure, read the appropriate sections before making your selection.

☒ Life sciences ☐ Behavioural & social sciences ☐ Ecological, evolutionary & environmental sciences

For a reference copy of the document with all sections, see nature.com/documents/nr-reporting-summary-flat.pdf

Life sciences study design

All studies must disclose on these points even when the disclosure is negative.

Sample size	No statistical method was used to pre-determine sample size.
Data exclusions	No data was excluded from the analyses.
Replication	Each experiment was repeated three times using three independent biological replicates. Each replicate generated reliable and similar result.
Randomization	For each experiment, samples (organoids or animals) were selected randomly from each experimental groups.
Blinding	Identical gains and exposure times were used during fluorescent imaging. For quantitative comparison through ImageJ or Angio tool, identical thresholds were applied. Blinding was not required to generate data from immunostaining.

Reporting for specific materials, systems and methods

We require information from authors about some types of materials, experimental systems and methods used in many studies. Here, indicate whether each material, system or method listed is relevant to your study. If you are not sure if a list item applies to your research, read the appropriate section before selecting a response.

Materials & experimental systems

n/a	Involved in the study
☐	☒ Antibodies
☐	☒ Eukaryotic cell lines
☒	☐ Palaeontology
☐	☒ Animals and other organisms
☒	☐ Human research participants
☒	☐ Clinical data

Methods

n/a	Involved in the study
☒	☐ ChIP-seq
☒	☐ Flow cytometry
☒	☐ MRI-based neuroimaging

Antibodies

Antibodies used	All antibodies are listed in Table 1 with their supplier name, catalogue/clone number and dilutions.
Validation	All the antibodies were validated for immunofluorescence staining with preliminary experiments. We examined all antibodies according to manuals, and got similar results with validation results on manufacturer's website or relevant citations.

Eukaryotic cell lines

Policy information about cell lines

Cell line source(s)	WTC11 human induced pluripotent stem cells were received from Bruce Conklin, Gladstone Institute of Cardiovascular Disease. H9 human embryonic stem cells were purchased from WiCell. H9-FP human embryonic stem cells were recieved from Andrew McMahon, University of Southern California.
Authentication	WTC11 is authenticated by Bruce Conklin, Gladstone Institute of Cardiovascular Disease.<br>H9 is authenticated by WiCell.<br>H9-FP is authenticated by Andrew McMahon, University of Southern California.
Mycoplasma contamination	All cell lines were tested negative for mycoplasma contamination.
Commonly misidentified lines (See ICLAC register)	No commonly misidentified cell line was used.

Animals and other organisms

Policy information about studies involving animals; ARRIVE guidelines recommended for reporting animal research

Laboratory animals	All animals were purchased from Jackson Laboratory or Charles River. Animal experiments were reviewed and approved by the Institutional Animal Care and Use Committees (IACUC) of Maine Medical Center and the University of Texas Southwestern Medical Center.
Wild animals	No wild animals were involved in this study.

Field-collected samples	No field-collected samples were involved in this study.
Ethics oversight	All animal experiments were reviewed and approved by the Institutional Animal Care and Use Committees (IACUC) of Maine Medical Center and the University of Texas Southwestern Medical Center.

Note that full information on the approval of the study protocol must also be provided in the manuscript.
